# Ancestry-Specific Hypothetical Genetic Feedback About Lung Cancer Risk in African American Individuals Who Smoke: Cognitive, Emotional, and Motivational Effects on Cessation

**DOI:** 10.3390/bs15070980

**Published:** 2025-07-19

**Authors:** Joel Erblich, Khin Htet, Camille Ragin, Elizabeth Blackman, Isaac Lipkus, Cherie Erkmen, Dina Bitterman

**Affiliations:** 1 Department of Psychology, Hunter College, City University of New York, New York, NY 10065, USA; 2 Cancer Prevention and Control Program, Fox Chase Cancer Center, Philadelphia, PA 19111, USA; 3 Department of Psychiatry, Duke University, Durham, NC 27708, USA; 4 Department of Thoracic Medicine and Surgery, Lewis Katz School of Medicine, Temple University, Philadelphia, PA 19122, USA

**Keywords:** genetic counseling, lung cancer, risk perceptions, health behavior, smoking, experiment

## Abstract

Genetic factors play an important role in the risk of developing lung cancer, a disease that disproportionately affects African American (AA) individuals who smoke. Accumulating evidence suggests that specific ancestry-informative genetic markers are predictive of lung cancer risk in AA individuals who smoke. Although testing for, and communication of, genetic risk to patients should impact health and screening, results are mixed. The goal of this study was to evaluate the effects of genetic risk communication that also included ancestry-specific risk information among African American individuals who smoke. Using an experimental design, African American individuals who smoke (n = 166) were assigned randomly to receive hypothetical genetic test results that indicated (1) low vs. high genetic risk for lung cancer (“Risk”) and (2) European vs. African Ancestry (“Ancestry”). We hypothesized that participants who had been told that they were both at high risk for lung cancer based on genetic markers prominent in African persons at risk of lung cancer, and that they have African ancestry, would exhibit increases in cognitive (perceived lung cancer risk), emotional (cancer worry and psychological distress), and motivational (motivation to quit smoking) factors shown to predict longer-term health behavior change. Results revealed significant and moderate-to-large effects of Risk for all outcomes. There was also a significant Ancestry effect on perceived lung cancer risk: increased risk perceptions among participants who learned that they have high African genetic heritage. Path analytic modeling revealed that cognitive and emotional factors mediated the effects of both Risk and Ancestry feedback on motivation to quit smoking. Findings further highlight the importance of incorporating ancestry-specific genetic risk information into genetic counseling sessions, especially in underserved populations, as doing so may impact key cognitive, emotional, and motivational factors critical to behavior change.

## 1. Introduction

African American (AA) individuals who smoke have long faced disproportionate rates of lung cancer incidence and mortality compared to White individuals who smoke (Cooley & Jennings-Dozier, 1998; Siegel et al., 2017). Recent data reveal promising drops in both incidence and mortality among AA individuals who smoke (Giaquinto et al., 2022), yet disparities remain (American Lung Association, 2022). In addition, although AA individuals smoke fewer cigarettes per day than White individuals (Haiman et al., 2006; Schwartz et al., 2009), they are less likely to stop smoking and are more likely to engage in deeper and longer inhalations (Berg et al., 2010; Giovino et al., 2004; Wassenaar et al., 2015). While systemic and socioeconomic barriers, including access to healthcare, contribute prominently to these disparities (Bonner et al., 2024; Cooley & Jennings-Dozier, 1998; Dawson & Fletcher, 2020), biological factors also play an important role. Specifically, several studies (Clark et al., 1996; Gandhi et al., 2009; Kabat et al., 1991) have raised the possibility that lung cancer disparities between AA and White individuals may be partially attributable to genetic factors related to poorer metabolism of tobacco-related carcinogens and increased addiction risk (Li et al., 2014; Schwartz et al., 2009). Moreover, AA individuals who smoke may have unique genetic cancer risk factors based on their ancestry, such that risk markers may be overrepresented in individuals with African genetic ancestry (Nair et al., 2022; Stepler et al., 2022). Thus, providing personalized genetic ancestry risk information may be more effective in encouraging cessation than providing traditional generic risk information. Indeed, such approaches have been gaining appeal more broadly in the field of precision medicine (Pereira et al., 2021) and highlight the potential utility of including personalized risk information in risk communication interventions. This work is particularly important given the historical underrepresentation of African Americans in genetic research and current efforts to reverse these trends (Gouveia et al., 2025). It is, of course, critical at the outset to emphasize the distinction between genetic ancestry, which reflects underlying genetic characteristics of one’s ancestors, and ‘race,’ which is a social construct (Duello et al., 2021). In the context of this field, for example, both European and African genetic ancestry exist in almost all racial groups in the United States due to well-established admixture (Aldrich et al., 2012; Aldrich et al., 2008). Genetic counselors stand at the front lines of risk communication and serve a critical role in discussing this nuanced personalized risk information with clients as part of shared decision-making (Roberts et al., 2020). Moreover, research has demonstrated that genetic counselors’ provision of personalized cancer genetic risk information, especially in underserved populations, increases knowledge critical to behavior change (Hurtado-de-Mendoza et al., 2017). The goal of the present study is to better understand factors that genetic counselors may find useful in encouraging behavior change among clients who smoke and are at risk for lung cancer.

Surprisingly little is known about the effectiveness of providing genetic risk information to patients to encourage behavior change, especially smoking cessation, with recent meta-analyses revealing little to no overall effects (Hollands et al., 2016; Marteau et al., 2010). The few experimental studies on receipt of lung cancer genetic risk information produced mixed effects on smoking cessation. Lerman et al. (1997) and McBride et al. (2010) identified only small and short-lived effects, with McBride et al. suggesting that generic risk information may have limited impact on behavior change without a more personalized risk profile. Consistent with this, a meta-analysis by French et al. (2017) found that while personalized risk information did not reliably increase healthy behaviors in general, effects were found for smoking cessation. A study by Lipkus et al. (2004) found that AA individuals who smoke often misinterpret generic risk information, highlighting the need for personalized risk communication. In a recent pilot study, Ramsey et al. (2021) reported an increased readiness to quit and a reduction in smoking amongst AA individuals who smoke who received personalized genetic risk information about lung cancer, COPD, and difficulty quitting, geared toward individuals with African ancestry. Findings supported the potential importance of personalized genetic risk information as a motivator for cessation. Ancestry-informative markers linked to risk variants have been identified for a number of diseases and conditions, including hypertension (especially in individuals who smoke) (Sung et al., 2018), fatty liver (Cavalcante et al., 2022), alcohol dependence (Sloan et al., 2018), and smoking-related lung morbidity (Aldrich et al., 2012). These studies raise the possibility that including ancestry-informative genetic information may make risk communications with patients more relevant, and hence, more effective.

The literature lacks clarity of potential mechanisms underlying the effects of personalized genetic risk communication on motivation to change behavior, which is crucial for improving risk communication interventions. Understanding mechanisms underlying motivation to quit would enhance strategies to tailor and further personalize risk communications to improve the impact of informational interventions and ultimately improve cessation.

Classic psychological models of behavior change have highlighted the importance of motivation to change, as well as attendant cognitive and emotional factors, in encouraging healthy behaviors. For example, both the Theory of Planned Behavior (Ajzen, 1991), as well as the Transtheoretical Model of behavioral change (Prochaska et al., 2009) focus on increasing motivation to change. The Health Belief Model (Rosenstock, 1966) posits that the perceived risk of negative outcomes (cognitive factor) is a key predictor of behavior change and an important target in risk communication interventions. Finally, Tallis and Eysenck (1994) highlight that emotional factors, such as worry, also influence behavior change. Despite the importance of integrating cognitive, emotional, and motivational factors in the study of smoking behavior, few studies have done so. Some exceptions include Magnan et al. (2009), who found that both perceived risk and worry predicted motivation to quit following repeated exposure to information about the negative consequences of smoking. Diaz et al. (2020) reported links between perceived lung cancer risk and worry about lung cancer, but did not assess cessation. Kaufman et al. (2018) found that while neither perceived risk nor cancer worry predicted smoking cessation, their interaction approached significance. In another study, Erblich et al. (2000b) found that perceived risk and cancer-related distress were predictive of breast cancer screening behavior. In a meta-analysis, Sheeran et al. (2014) found that perceived risk increased intentions to change health-related behaviors, especially in the presence of negative emotions, highlighting the importance of modeling the combined impact of cognitive and emotional factors in motivating behavior change.

To our knowledge, research examining the cognitive, emotional, and motivational effects of ancestry-specific genetic risk communication in AA individuals who smoke is lacking. To that end, we conducted an experimental study to investigate the effects of providing ancestry-specific (African vs. European ancestry) and lung cancer genetic risk (low vs. high) information on perceived risk, cancer worry, distress, and motivation to quit smoking among AA individuals who smoke. Because current clinical practice does not yet employ definitive personalized genetic risk information for lung cancer risk, our study employed hypotheticals, as described below (see Section 2 Methods). We hypothesized that communication of hypothetical genetic risk and ancestry would increase perceived lung cancer risk, cancer worry, general psychological distress, and motivation to quit smoking. We also predicted an interaction, with the greatest effects observed among individuals receiving high-risk and African ancestry information, thus increasing the personal relevance of the information. Furthermore, we hypothesized that increases in perceived risk, worry, and psychological distress would mediate the effects of genetic risk and ancestry communication on motivation to quit smoking.

## 2. Methods

*Participants*. African American individuals who smoke cigarettes (n = 166) were recruited. Our original strategy aimed to recruit individuals who smoke from community centers in the Greater New York City and Philadelphia regions by working with the Community Outreach Core of the Temple University/Fox Chase Cancer Center-Hunter College Collaborative Partnership to Advance Cancer Health Equity (CPACHE). We successfully recruited thirty-eight participants from community centers. However, due to the onset of the COVID-19 pandemic, we were forced to continue recruitment remotely, using Amazon M-Turk, through which we recruited the remaining one hundred twenty-eight participants. Criteria for participation included: (1) self-identification as Black/African American; (2) being aged 18 years or older; (3) self-identification as an individual who currently smokes at least five cigarettes per day on the average; (4) having smoked at least one hundred lifetime cigarettes; and (5) having no past or current history of a cancer diagnosis. A priori power analysis indicated that n = 146 would yield power of 0.85 to detect moderate effect sizes (f = 0.25) at a two-tailed alpha level of 0.05. To allow for missingness, we recruited 166 participants and were fortunate not to have missing data. The sample consisted of 102 women (61.4%) and 64 men (38.6%), with a mean age of 41.1 (SD = 14.4). Due to an error in the M-Turk platform, data on education level were not available.

*Procedures*. All procedures were reviewed and received the necessary ethics approvals from the Institutional Review Boards of both Hunter College (#2019-0472) and the Fox Chase Cancer Center (#18-4004). All participants provided informed consent prior to participation. The trial was pre-registered on clinicaltrials.gov (ID# NCT04084561). Using the REDCap randomization module, participants were doubly randomly assigned to receive either Low vs. High genetic risk and either European vs. African genetic ancestry feedback, yielding four groups, in a 2 × 2 factorial design: Low Risk/European Ancestry (n = 40), High Risk/European ancestry (n = 43), Low Risk/African Ancestry (n = 42), and High Risk/African ancestry (n = 41). Study personnel were not blinded. All participants completed questionnaires immediately before and after reading a brief “personalized” report that described genetic testing and provided them with hypothetical results of two tests: one assessing the risk of developing lung cancer and the other assessing genetic ancestry. The report was modeled after previous work in this area (Lipkus et al., 2014) and included background information about genetic risk, indicating that: (1) genetic factors can play a role in lung cancer risk; and (2) individuals who have genetic African Ancestry may be at high risk for lung cancer. The final page of the report offered hypothetical results of such genetic testing, which varied based on the randomization scheme. “Low Risk” participants received the following feedback: “You are at approximately 7–10% risk of developing lung cancer in your life if you continue smoking. This risk is essentially equal to the general population of smokers.” “High Risk” participants received the following feedback: “You are at approximately 50–80% risk of developing lung cancer in your life if you continue smoking. This risk is substantially higher than the general population of smokers.” We chose these risk levels to mirror other known genetic cancer risks (i.e., BRCA1/2 carrier status) that have been well-publicized in both the scientific and popular literature (Antoniou et al., 2003). In addition, those randomized to the “European Ancestry” condition received the following genetic feedback: “Your genetic ancestry panel indicates that your ancestry is approximately 10% African and 90% European origin,” while those in the “African ancestry” condition received the following genetic feedback: “Your genetic ancestry panel indicates that your ancestry is approximately 90% African and 10% European origin.”. We hypothesized that the more personalized combination of receiving high-risk and African ancestry information as described in the report would lead to the greatest changes in perceived risk, distress, and motivation to quit. We note that because ancestry-informative markers of lung cancer risk are still being identified and validated, they are not yet being communicated to African American patients in routine clinical practice. Thus, to maximize experimental flexibility, “hypothetical” results were given. Providing hypothetical feedback has been well-validated in previous work as a feasible, flexible, and valid approach to the experimental study of risk communication [e.g., Lipkus et al. (2014)]. Finally, we included six brief informational questions as an attention check to make sure that participants attended to the report, and to help identify automated responses (e.g., ‘bots’). All participants successfully passed the attention check items.

*Measures*. Participants completed the 6-item Fagerstrom Test of Nicotine Dependence [FTND; Heatherton et al. (1991)], as well as the 8-item Subjective Numeracy Scale [SNS; Fagerlin et al. (2007)] prior to the experimental manipulation, as previous research has suggested that numeracy can influence risk perceptions (Reyna et al., 2009). Next, participants completed five key study measures immediately before and after the genetic risk report: First, we assessed perceived absolute lung cancer risk, measured as a single 0 (“no chance at all”) to 10 (“for sure will get it”) Likert-type scale. Next, we assessed perceived relative lung cancer risk, measured as a single item assessing perceived lifetime risk of developing lung cancer “compared to other smokers your age,” using a 5-item face-valid Likert-type scale, ranging from “much less likely” to “much more likely.” We have successfully employed brief face-valid measures of perceived risk in our previous work (DiLorenzo et al., 2006; Erblich et al., 2000a). Participants then completed the 4-item Cancer Worry Scale [e.g., “If you were given the actual genetic information you just heard, how much would you be worried about your chances of getting lung cancer?”] (Andersen et al., 2003), as well as an 18-item version of the Brief Symptom Inventory [BSI-18; Derogatis (2001)], a rapid assessment of general psychological distress (e.g., fearful, depressed, tense). For this brief measure, we modified the response window to reflect how the participant would feel after receiving the hypothetical information. Participants completed the 7-item Motivation to Stop Scale [MTSS; (Kotz et al., 2013)] as an index of motivation to quit smoking which has been shown to prospectively predict actual quit attempts (Hummel et al., 2017, 2018). Pre-report administrations of perceived risk, cancer worry, psychological distress, and motivation to quit included instructions to participants to evaluate their current feelings, whereas post-report administrations instructed participants to indicate how they would feel if they had actually received the genetic risk information presented in the report. All questionnaires were administered in fixed order for all participants.

*Data Analysis*. Preliminary analyses revealed that the community-based participants were significantly older (M = 62.5, SD = 10.3) than the M-Turk participants (35.0, SD = 8.1), *t*(164) = 17.3, *p* < 0.001 and had significantly lower levels of nicotine dependence on the FTND (M = 2.4, SD = 1.9) than M-Turk participants (M = 4.3, SD = 1.8), *t*(164) = 5.7, *p* < 0.001. Thus, to be conservative, we included age and FTND as covariates in the primary analyses. SNS scores were not related to any of the study variables, and thus were excluded from subsequent analyses. To address the primary study hypotheses, we conducted a series of between-within 2 (“Risk”—Low vs. High Genetic Risk) × 2 (“Ancestry”—European vs. African Ancestry) × 2 (“Time”—Pre-Exposure vs. Post-Exposure) ANOVAs for each of the five study variables: perceived absolute lung cancer risk, perceived relative lung cancer risk, cancer worry, general psychological distress, and motivation to quit. Planned simple-effects comparisons were Bonferroni corrected to control for Type I error.

We also conducted path analyses to model direct and indirect effects of risk and ancestry feedback on motivation to quit smoking. We calculated residualized and standardized change scores [i.e., standardized change scores from pre- to post-exposure corrected for pre-exposure levels (Castro-Schilo & Grimm, 2018)] for motivation to quit (outcome), as well as perceived risk, cancer worry, and general psychological distress (mediators). Using SPSS PROCESS 4.1 (Hayes, 2022), we estimated direct and indirect effects of Risk and Ancestry on changes in motivation to quit via the key mediators, perceived risk, cancer worry, and general psychological distress. We estimated bootstrapped 95% confidence intervals (k = 20,000 resamples) to evaluate the significance of the indirect effects. In addition to simple mediation, we also explored two more complex serial mediation models, in which effects on motivation to quit were mediated by: (1) perceived risk through increases in cancer worry, and (2) perceived risk through increases in general psychological distress. To avoid redundancy, we did not run parallel mediation models, as we were already running simple mediation models for the individual predictors. In all analyses, FTND and age were included as covariates, but results were the same with or without inclusion.

## 3. Results

**Figure 1:**
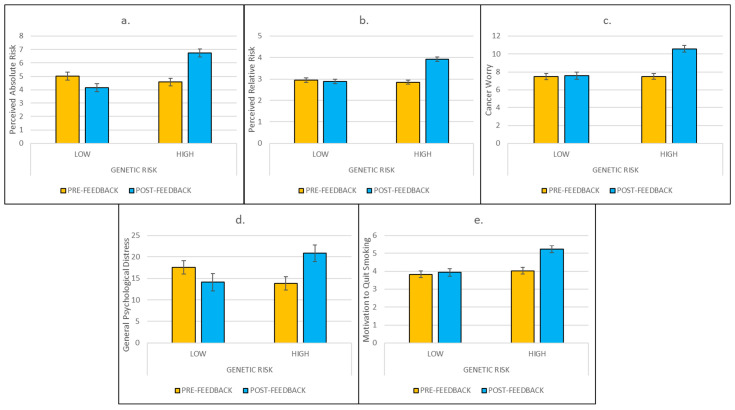
Risk × Time effects of hypothetical genetic risk feedback on perceived absolute risk, perceived relative risk, cancer worry, general psychological distress, and motivation to quit smoking (panels (**a**–**e**), respectively). Note: Effects depicted in all panels are significant (*p*’s < 0.001). Alt Text: Five graphs depicting significant increases in perceived absolute risk, perceived relative risk, cancer worry, general psychological distress, and motivation to quit smoking among smokers who received high genetic risk feedback, but not among smokers receiving low genetic risk.

*Effects of lung cancer risk feedback on perceived lung cancer risk*. Effects of genetic risk feedback are depicted in Figure 1. Consistent with study hypotheses, findings revealed significant Risk × Time interactions for both perceived absolute (F[1,160] = 49.70, *p* < 0.001, η^2^ = 0.24) and relative (F[1,160] = 47.89, *p* < 0.001, η^2^ = 0.23) risks of lung cancer. In a series of Bonferroni-corrected planned comparisons, participants in the hypothetical Low-Risk Group exhibited significantly lower perceived absolute risk following feedback (mean change = −0.86; 95% CI: −1.45, −0.26; *p* < 0.005), whereas participants in the hypothetical High-Risk Group exhibited significantly higher perceived absolute risk following feedback (mean change = 2.16; 95% CI: 1.57, 2.76; *p* < 0.001). Participants in the hypothetical Low-Risk Group did not exhibit significant changes in perceived relative risk following feedback (mean change = −0.08; 95% CI: −0.31, 0.15; *p* < 0.511). Participants in the hypothetical High-Risk Group, however, exhibited significantly higher perceived relative risk following feedback (mean change = 1.07; 95% CI: 0.84, 1.30; *p* < 0.001).

*Effects of lung cancer risk feedback on cancer worry and general psychological distress*. We found significant Risk × Time interactions for both cancer worry (F[1,160] = 43.79, *p* < 0.001, η^2^ = 0.21) and BSI (F[1,160] = 24.19, *p* < 0.001, η^2^ = 0.13). Bonferroni-corrected planned comparisons revealed that participants in the hypothetical Low-Risk Group did not exhibit significant changes in cancer worry (mean change = 0.07; 95% CI: −0.57, 0.72; *p* < 0.821), but did exhibit significant decreases in general psychological distress (mean change = −3.54; 95% CI: −6.58, −0.50; *p* < 0.023), following feedback. Participants in the hypothetical High-Risk Group, however, exhibited both significantly higher cancer worry (mean change = 3.11; 95% CI: 2.47, 3.74; *p* < 0.001) and general psychological distress (mean change = 7.13; 95% CI: 4.12, 10.13; *p* < 0.001) following feedback.

*Effects of lung cancer risk feedback on motivation to quit smoking*. We found a significant Risk × Time interaction effect on motivation to quit (F[1,160] = 32.10, *p* < 0.001, η^2^ = 0.17). Bonferroni-corrected planned comparisons revealed that participants in the hypothetical Low-Risk Group did not exhibit significant changes in motivation to quit (mean change = 0.10; 95% CI: −0.18, 0.37; *p* < 0.495), whereas participants in the hypothetical High-Risk Group exhibited significantly higher motivation to quit following feedback (mean change = 1.21; 95% CI: 0.94, 1.48; *p* < 0.001).

**Figure 2:**
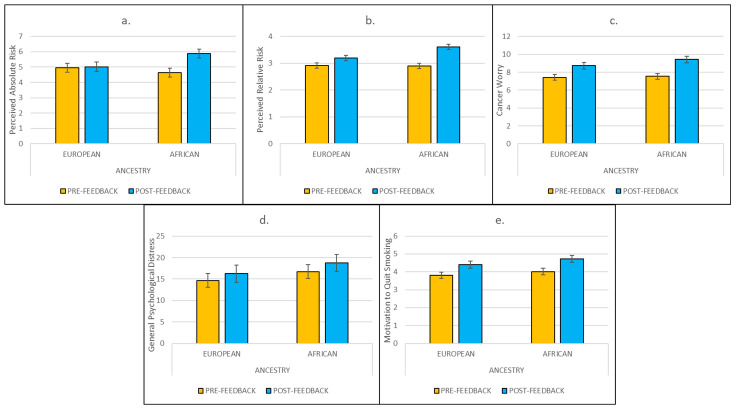
Ancestry × Time effects of hypothetical genetic ancestry feedback on perceived absolute risk, perceived relative risk, cancer worry, general psychological distress, and motivation to quit smoking (panels (**a**–**e**), respectively). Note: Only effects depicted in panels (**a**,**b**) (*p*’s < 0.005 and 0.019, respectively) are significant. Alt Text: Five graphs depicting increases in perceived absolute risk, perceived relative risk, cancer worry, general psychological distress, and motivation to quit smoking among smokers who received African ancestry feedback. Effects of ancestry are only significant for perceived absolute risk and perceived relative risk.

*Effects of ancestry feedback on perceived lung cancer risks, worry and psychological distress*. Effects of hypothetical ancestry feedback are depicted in Figure 2. Consistent with the study hypotheses, findings revealed significant Ancestry × Time interactions for both perceived absolute (F[1,160] = 7.98, *p* < 0.005, η^2^ = 0.05) and relative (F[1,160] = 5.62, *p* < 0.019, η^2^ = 0.03) lung cancer risks. In a series of Bonferroni-corrected planned comparisons, participants in the hypothetical European Ancestry Group did not exhibit significant changes in perceived absolute risk following feedback (mean change = 0.06; 95% CI: −0.54, 0.66; *p* < 0.842), whereas participants in the hypothetical African Ancestry Group exhibited significantly higher perceived absolute risk following feedback (mean change = 1.24; 95% CI: 0.64, 1.84; *p* < 0.001). With respect to relative risk, both the hypothetical European (mean change = 0.30; 95% CI: 0.07, 0.53; *p* < 0.012) and hypothetical African (mean change = 0.70; 95% CI: 0.46, 0.93; *p* < 0.001) Ancestry Groups exhibited increases following feedback. There were no significant Ancestry × Time effects on cancer worry (*p* < 0.276), general psychological distress (*p* < 0.997), or motivation to quit (*p* < 0.612). None of the three-way Risk × Ancestry × Time interaction effects were significant for any of the study outcomes (*p*’s > 0.210), suggesting that there were no multiplicative effects of genetic risk and hypothetical ancestry feedback.

**Figure 3:**
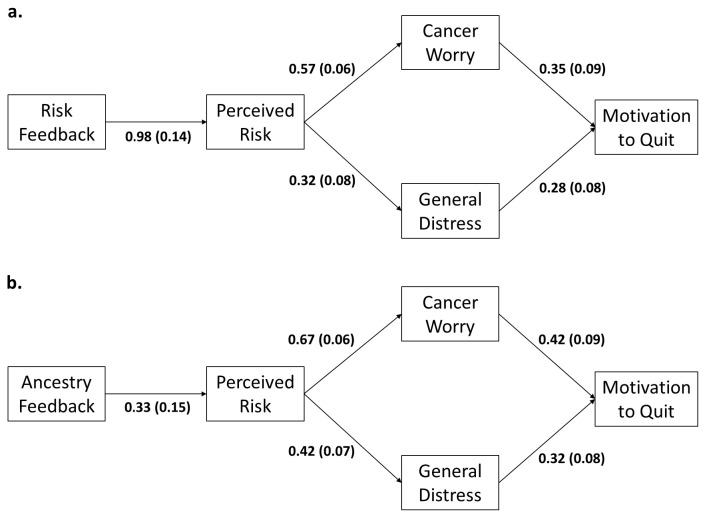
Path models depicting serial mediation of effects of hypothetical risk (**a**) and ancestry (**b**) feedback on motivation to quit smoking. Displayed are standardized regression coefficients and standard errors (all *p*’s < 0.05). Alt Text: Two diagrams depicting the significant effects of risk and ancestry feedback on perceived risk, which in turn predicts cancer worry and general distress, which in turn predict increases in motivation to quit smoking.

*Indirect effects of risk feedback on motivation to quit smoking*. We conducted path analyses modeling direct and indirect effects of risk and hypothetical ancestry feedback on motivation to quit smoking, with perceived risk, cancer worry, and psychological distress as mediators. Because results were virtually identical to perceived absolute risk, we decided not to report the perceived relative risk mediational models for brevity. Parameter estimates for the models are summarized in Table 1. Findings revealed significant indirect effects of Risk feedback on motivation to quit smoking through increased perceived risk, cancer worry, and general psychological distress. In addition, we identified a significant indirect effect of hypothetical Ancestry feedback on motivation to quit smoking through increases in perceived risk. In all cases, bootstrapped confidence intervals did not include zero. Due to the lack of direct effects of hypothetical Ancestry feedback on cancer worry or general psychological distress, we did not observe significant indirect effects via these mediators on motivation to quit. The serial mediational models revealed significant indirect effects for both Risk and hypothetical Ancestry feedback, such that increased perceived risk predicted heightened cancer worry and general psychological distress, which, in turn, predicted increased motivation to quit smoking (see Table 1 and Figure 3). To further explore the directionality of effects, we reran the models with the mediators in the reverse order; however, the models were found to be non-significant. Finally, because none of the three-way interactions above were significant for any of the outcomes, we did not explore path models for these effects.

## 4. Discussion

Our study demonstrates that communicating hypothetical personalized genetic risk and ancestry information significantly increased perceived risk of lung cancer, albeit as main effects, rather than interactions. This indicates that the provision of high-risk and African ancestry information produces independent and additive, but not multiplicative, increases in perceived risk, with stronger effects of genetic risk than ancestry. Furthermore, providing hypothetical genetic lung cancer risk feedback led to increases in cancer worry, general psychological distress, and motivation to quit smoking. Path analyses revealed significant indirect effects, which indicated that personalized genetic lung cancer risk and ancestry feedback increased perceived lung cancer risk, which, in turn, predicted increases in cancer worry and general psychological distress, ultimately leading to increased motivation to quit smoking. Thus, our findings provided support that genetic feedback influences key cognitive and emotional mediators of motivation to change.

Our results are consistent with some prior research that highlights the need for greater tailoring when communicating genetic risk to promote behavior change. McBride et al. (2010) argued cogently for the importance of providing more personalized risk information. In this study, we framed genetic risk by indicating that the risk factors were particularly salient among AA individuals who smoke, which may account for the findings we observed. French et al. (2017), while reporting mixed results with respect to the effects of personalized genetic information, suggest that the strongest signal came from studies of lung cancer risk. Finally, the present findings build upon the pilot work of Ramsey et al. (2021) who found initial support for the effects of the provision of personalized genetic risk information on smoking outcomes. We acknowledge, however, that although both were significant, the effects of Risk were considerably stronger than the effects of Ancestry. Thus, while the provision of ancestry information alone increased cognitive, emotional, and motivational outcomes, the effects were more limited.

In addition to demonstrating the effects of personalized genetic risk information, findings elucidated potentially key cognitive and emotional pathways that underlie effects on the motivation to quit smoking. Thus, this study, using hypothetical genetic risk and ancestry information, offers proof of concept that personalized genetic risk communication relevant to lung cancer can enhance motivation to quit, while also identifying possible cognitive and emotional targets for intervention to further enhance the efficacy of effects going forward. These targets are critical as researchers move closer to the clinical validation of personalized, ancestry-informed genetic risk factors, which are likely to become routine in cancer risk communication. We also note that although our focus was on lung cancer risk and motivation to quit smoking, the results suggest that communication of personalized genetic risk information about other diseases (e.g., breast cancer and cardiovascular disease) may encourage appropriate health and screening behavior (e.g., routine mammography, cholesterol testing, improved diet, and exercise), although future research is needed.

Key practice implications of this study include the potential utility of providing personalized genetic and ancestry risk information to AA individuals who smoke, along with understanding the mechanisms underlying their potential utility in promoting motivation to quit smoking. Moreover, the significant indirect effects identified both cognitive (perceived risk of lung cancer) and emotional (cancer worry and general psychological distress) factors that play a role in motivating smoking cessation. Although the information in the present study was hypothetical and thus preclinical in nature, the implications for genetic counseling are evident; the provision of personalized ancestry- and genetic-risk information, especially in underserved patients, might be a useful approach in counseling patients about their risk and guiding them through health decision-making. Leveraging precision medicine in genetic counseling continues to be an important tool in maximizing the impact of counseling. As the use of polygenic risk scores becomes increasingly common in risk assessment for a number of adverse health outcomes in both AA and White individuals, including substance abuse [e.g., (Hartwell et al., 2022; Thomas et al., 2021)], the importance of competently and sensitively communicating personalized risk becomes even more critical.

It is worth noting that while many studies have identified positive relationships between perceived risk, worry, and motivation to change behavior, others have found a reverse effect. For example, Carcioppolo (2016) found that high levels of perceived risk could lead to lower motivation to change cancer-protective behaviors, especially when combined with high levels of fatalism. It is possible that very high levels of perceived risk may serve to increase fatalistic attitudes and inhibit behavior change. Similarly, high levels of worry or psychological distress associated with receiving risk information may inhibit motivation for behavior change by facilitating avoidance.

Consistent with this possibility, Lerman et al. (1994) and Kash et al. (1992) found that high levels of cancer worry were associated with poorer compliance with breast cancer screening. Emanuel et al. (2015) found that almost 40% of a nationally representative sample reported a preference not to learn their risk of developing cancer, highlighting the potentially influential role of avoidance of risk communication. In concert with the present study’s findings, we propose that moderate increases in perceived risk, worry, and distress can be facilitative, whereas higher levels may inhibit desired behaviors. Relevant to counseling practice, genetic counseling might need to be keyed to a “sweet spot,” activating rather than inhibiting behavior change. It is possible that the moderate increases in perceived risk, worry, and distress elicited in this study were due to providing hypothetical rather than actual personalized risk information. Studies that employ actual personalized information might benefit from caution over triggering perceived risk, worry, and distress at levels that potentially place the patient at risk of avoidance. Indeed, it is tempting to speculate that some of the negative findings of the effects of risk communication in the literature may be due to overly heightened risk perceptions and/or distress that may have had the unintended consequence of inhibiting target health and screening behaviors. For this reason, it is all the more critical to assess the underlying mechanisms of change in addition to the actual effects when evaluating the efficacy of risk communication interventions.

This study has several key limitations. First and foremost, the goal of achieving tighter experimental control was counterbalanced by providing hypothetical feedback. Although prior work (Lipkus et al., 2014) has shown that hypothetical risk communication is a useful method to evaluate effects, eventual replication in clinical settings with actual risk information is warranted. It is possible that actual genetic results might provoke different emotional or motivational responses.

Another methodological limitation was the inclusion of both in-person and online recruitment in this study. Although we addressed the differences in data sources in our statistical analyses, it remains unknown whether or not there might be differences in content engagement or generalizability. Perhaps most importantly, to maintain rigorous experimental control, we employed very structured, yet somewhat simplistic, forms of genetic risk and ancestry information. As our understanding of genetic risk and ancestry evolves, patients attending genetic counseling will be exposed to more complex and nuanced information, rather than simple “high vs. low” risk, or “African vs. European” ancestry, and studies will need to evaluate methods to most effectively and sensitively communicate this information. Indeed, a critical concern with personalized genetic medicine, especially in vulnerable populations, is to make sure that ethical guidelines are adhered to, minimizing stigma, reflecting on the inherent limitations of this work, and clearly distinguishing between race and genetic ancestry, as discussed above. In addition, in this cross-sectional study, the proximal outcome was motivation to quit smoking. Although motivation to quit is a strong predictor of actual quit attempts as well as ultimate quit success (Ajzen, 1991; Klemperer et al., 2020), many other factors contribute to an individual who smoke’s decision to quit. Future prospective studies should follow individuals who smoke longitudinally to definitively evaluate the effects of providing personalized ancestry-informed genetic risk on smoking cessation outcomes. Finally, we relied on responses to self-report questionnaires, which are subject to inherent limitations, including absent-minded responding and imperfect psychometric properties. To minimize the latter concern, we made an effort to include well-validated instruments in this study, as described above.

Of course, although genetic factors play an important role in risk, they are but one of many behavioral, social, cultural, and environmental factors that contribute (Bonner et al., 2024). This study did not address those factors, and the effects of genetic factors per se need to be considered in the context of broader social determinants of health. Counselors should consider all sources of risk when counseling their patients and be mindful of the important distinction between the concept of genetic ancestry and the social construct of race as mentioned above. Overall, the present study provides experimental evidence that the communication of personalized genetic information about lung cancer can potentially increase motivation to quit smoking by enhancing perceived risk, which, in turn, is associated with higher levels of cancer worry and psychological distress.

## Figures and Tables

**Table 1:** Standardized direct (asymptotic 95% CIs) and indirect effects (bootstrapped 95% CIs) of hypothetical Risk and Ancestry feedback on motivation to quit smoking. Bolded Effects are statistically significant.

Mediator	Hypothetical Risk Feedback		Hypothetical Ancestry Feedback	
	Direct Effect	Indirect Effect	Direct Effect	Indirect Effect
Simple Mediation				
PR⟶MQ	**0.60 (0.29, 0.92)**	**0.24 (0.10, 0.42)**	−0.04 (−0.33, 0.25)	**0.13 (0.01, 0.28)**
CW⟶MQ	**0.49 (0.19, 0.78)**	**0.36 (0.18, 0.60)**	0.02 (−0.25, 0.29)	0.07 (−0.08, 0.22)
GD⟶MQ	**0.62 (0.34, 0.91)**	**0.22 (0.10, 0.36)**	0.09 (−0.19, 0.37)	0.00 (−0.14, 0.12)
Serial Mediation *				
PR⟶CW⟶MQ	**0.47 (0.16, 0.78)**	**0.20 (0.07, 0.36)**	−0.01 (−0.28, 0.26)	**0.09 (0.01, 0.21)**
PR⟶GD⟶MQ	**0.50 (0.20, 0.81)**	**0.09 (0.03, 0.17)**	0.00 (−0.27, 0.28)	**0.05 (0.01, 0.10)**

Note: PR = Perceived Absolute Risk, CW = Cancer Worry, GD = General Psychological Distress, MQ = Motivation to Quit. * Depicted in Figure 3.

## Data Availability

Summary data for meta-analyses will be provided upon request. Data from this manuscript have been presented previously at the 15th AACR Conference on the Science of Cancer Health Disparities in Racial/Ethnic Minorities and the Medically Underserved, Philadelphia, PA, September 2022.

## References

[B1-behavsci-15-00980] Ajzen I. (1991). The theory of planned behavior. Organizational Behavior and Human Decision Processes.

[B2-behavsci-15-00980] Aldrich M. C., Kumar R., Colangelo L. A., Williams L. K., Sen S., Kritchevsky S. B., Meibohm B., Galanter J., Hu D., Gignoux C. R., Liu Y., Harris T. B., Ziv E., Zmuda J., Garcia M., Leak T. S., Foreman M. G., Smith L. J., Fornage M., Gorlova O. Y. (2012). Genetic ancestry-smoking interactions and lung function in African Americans: A cohort study. PLoS ONE.

[B3-behavsci-15-00980] Aldrich M. C., Selvin S., Hansen H. M., Barcellos L. F., Wrensch M. R., Sison J. D., Quesenberry C. P., Kittles R. A., Silva G., Buffler P. A., Seldin M. F., Wiencke J. K. (2008). Comparison of statistical methods for estimating genetic admixture in a lung cancer study of African Americans and Latinos. American Journal of Epidemiology.

[B4-behavsci-15-00980] American Lung Association (2022). State of lung cancer 2022.

[B5-behavsci-15-00980] Andersen M. R., Smith R., Meischke H., Bowen D., Urban N. (2003). Breast cancer worry and mammography use by women with and without a family history in a population-based sample. Cancer Epidemiol Biomarkers Prev.

[B6-behavsci-15-00980] Antoniou A., Pharoah P. D., Narod S., Risch H. A., Eyfjord J. E., Hopper J. L., Loman N., Olsson H., Johannsson O., Borg Å., Pasini B., Radice P., Manoukian S., Eccles D., Tang N., Olah E., Anton-Culver H., Warner E., Lubinski J., Easton D. F. (2003). Average risks of breast and ovarian cancer associated with BRCA1 or BRCA2 mutations detected in case Series unselected for family history: A combined analysis of 22 studies. American Journal of Human Genetics.

[B7-behavsci-15-00980] Berg J. Z., Mason J., Boettcher A. J., Hatsukami D. K., Murphy S. E. (2010). Nicotine metabolism in African Americans and European Americans: Variation in glucuronidation by ethnicity and UGT2B10 haplotype. The Journal of Pharmacology and Experimental Therapeutics.

[B8-behavsci-15-00980] Bonner S. N., Curley R., Love K., Akande T., Akhtar A., Erhunmwunsee L. (2024). Structural racism and lung cancer risk: A scoping review. JAMA Oncology.

[B9-behavsci-15-00980] Carcioppolo N. (2016). When does perceived susceptibility to skin cancer influence indoor tanning? The moderating role of two risk perception beliefs. Journal of Health Communication.

[B10-behavsci-15-00980] Castro-Schilo L., Grimm K. J. (2018). Using residualized change versus difference scores for longitudinal research. Journal of Social and Personal Relationships.

[B11-behavsci-15-00980] Cavalcante L. N., Porto J., Mazo D., Longatto-Filho A., Stefano J. T., Lyra A. C., Carrilho F. J., Reis R. M., Alves V. A., Sanyal A. J., Oliveira C. P. (2022). African genetic ancestry is associated with lower frequency of PNPLA3 G allele in non-alcoholic fatty liver in an admixed population. Annals of Hepatology.

[B12-behavsci-15-00980] Clark P. I., Gautam S., Gerson L. W. (1996). Effect of menthol cigarettes on biochemical markers of smoke exposure among black and white smokers. Chest.

[B13-behavsci-15-00980] Cooley M. E., Jennings-Dozier K. (1998). Lung cancer in African Americans. A call for action. Cancer Practice.

[B14-behavsci-15-00980] Dawson D. B., Fletcher T. L. (2020). The influence of racial/ethnic discrimination experiences on cigarette craving for African American and hispanic smokers. Journal of Racial and Ethnic Health Disparities.

[B15-behavsci-15-00980] Derogatis L. (2001). Brief symptom inventory (BSI) 18: Administration, scoring, and procedures manual.

[B16-behavsci-15-00980] Diaz D., Fix B., Caruso R., Bansal-Travers M., O’Connor R. J. (2020). Worry about lung cancer is related to numeracy and risk perception of diseases associated with smoking. American Journal of Health Education.

[B17-behavsci-15-00980] DiLorenzo T. A., Schnur J., Montgomery G. H., Erblich J., Winkel G., Bovbjerg D. H. (2006). A model of disease-specific worry in heritable disease: The influence of family history, perceived risk and worry about other illnesses. Journal of Behavioral Medicine.

[B18-behavsci-15-00980] Duello T. M., Rivedal S., Wickland C., Weller A. (2021). Race and genetics versus ‘race’ in genetics: A systematic review of the use of African ancestry in genetic studies. Evolution, Medicine, and Public Health.

[B19-behavsci-15-00980] Emanuel A. S., Kiviniemi M. T., Howell J. L., Hay J. L., Waters E. A., Orom H., Shepperd J. A. (2015). Avoiding cancer risk information. Social Science & Medicine.

[B20-behavsci-15-00980] Erblich J., Bovbjerg D. H., Norman C., Valdimarsdottir H. B., Montgomery G. H. (2000a). It won’t happen to me: Lower perception of heart disease risk among women with family histories of breast cancer. Preventive Medicine.

[B21-behavsci-15-00980] Erblich J., Bovbjerg D. H., Valdimarsdottir H. B. (2000b). Psychological distress, health beliefs, and frequency of breast self-examination. Journal of Behavioral Medicine.

[B22-behavsci-15-00980] Fagerlin A., Zikmund-Fisher B. J., Ubel P. A., Jankovic A., Derry H. A., Smith D. M. (2007). Measuring numeracy without a math test: Development of the Subjective Numeracy Scale. Medical Decision Making.

[B23-behavsci-15-00980] French D. P., Cameron E., Benton J. S., Deaton C., Harvie M. (2017). Can communicating personalised disease risk promote healthy behaviour change? A systematic review of systematic reviews. Annals of Behavioral Medicine.

[B24-behavsci-15-00980] Gandhi K. K., Foulds J., Steinberg M. B., Lu S. E., Williams J. M. (2009). Lower quit rates among African American and Latino menthol cigarette smokers at a tobacco treatment clinic. International Journal of Clinical Practice.

[B25-behavsci-15-00980] Giaquinto A. N., Miller K. D., Tossas K. Y., Winn R. A., Jemal A., Siegel R. L. (2022). Cancer statistics for African American/Black People 2022. CA: A Cancer Journal for Clinicians.

[B26-behavsci-15-00980] Giovino G. A., Sidney S., Gfroerer J. C., O’Malley P. M., Allen J. A., Richter P. A., Cummings K. M. (2004). Epidemiology of menthol cigarette use. Nicotine & Tobacco Research.

[B27-behavsci-15-00980] Gouveia M. H., Meeks K. A. C., Borda V., Leal T. P., Kehdy F. S. G., Mogire R., Doumatey A. P., Tarazona-Santos E., Kittles R. A., Mata I. F., O’cOnnor T. D., Adeyemo A. A., Shriner D., Rotimi C. N. (2025). Subcontinental genetic variation in the All of Us Research Program: Implications for biomedical research. American Journal of Human Genetics.

[B28-behavsci-15-00980] Haiman C. A., Stram D. O., Wilkens L. R., Pike M. C., Kolonel L. N., Henderson B. E., Le Marchand L. (2006). Ethnic and racial differences in the smoking-related risk of lung cancer. New England Journal of Medicine.

[B29-behavsci-15-00980] Hartwell E. E., Merikangas A. K., Verma S. S., Ritchie M. D., Regeneron Genetics C., Kranzler H. R., Kember R. L. (2022). Genetic liability for substance use associated with medical comorbidities in electronic health records of African-and European-ancestry individuals. Addiction Biology.

[B30-behavsci-15-00980] Hayes A. F. (2022). Introduction to mediation, moderation, and conditional process analysis.

[B31-behavsci-15-00980] Heatherton T. F., Kozlowski L. T., Frecker R. C., Fagerstrom K. O. (1991). The fagerstrom test for nicotine dependence: A revision of the fagerstrom tolerance questionnaire. British Journal of Addiction.

[B32-behavsci-15-00980] Hollands G. J., French D. P., Griffin S. J., Prevost A. T., Sutton S., King S., Marteau T. M. (2016). The impact of communicating genetic risks of disease on risk-reducing health behaviour: Systematic review with meta-analysis. BMJ.

[B33-behavsci-15-00980] Hummel K., Brown J., Willemsen M. C., West R., Kotz D. (2017). External validation of the Motivation To Stop Scale (MTSS): Findings from the International Tobacco Control (ITC) Netherlands Survey. European Journal of Public Health.

[B34-behavsci-15-00980] Hummel K., Candel M., Nagelhout G. E., Brown J., van den Putte B., Kotz D., Willemsen M. C., Fong G. T., West R., de Vries H. (2018). Construct and predictive validity of three measures of intention to quit smoking: Findings from the International Tobacco Control (ITC) Netherlands Survey. Nicotine & Tobacco Research.

[B35-behavsci-15-00980] Hurtado-de-Mendoza A., Jackson M. C., Anderson L., Sheppard V. B. (2017). The role of knowledge on genetic counseling and testing in black cancer survivors at increased risk of carrying a BRCA1/2 mutation. Journal of Genetic Counseling.

[B36-behavsci-15-00980] Kabat G. C., Morabia A., Wynder E. L. (1991). Comparison of smoking habits of blacks and whites in a case-control study. American Journal of Public Health.

[B37-behavsci-15-00980] Kash K. M., Holland J. C., Halper M. S., Miller D. G. (1992). Psychological distress and surveillance behaviors of women with a family history of breast cancer. JNCI Journal of the National Cancer Institute.

[B38-behavsci-15-00980] Kaufman A. R., Dwyer L. A., Land S. R., Klein W. M. P., Park E. R. (2018). Smoking-related health beliefs and smoking behavior in the National Lung Screening Trial. Addictive Behaviors.

[B39-behavsci-15-00980] Klemperer E. M., Mermelstein R., Baker T. B., Hughes J. R., Fiore M. C., Piper M. E., Schlam T. R., Jorenby D. E., Collins L. M., Cook J. W. (2020). Predictors of smoking cessation attempts and success following motivation-phase interventions among people initially unwilling to quit smoking. Nicotine & Tobacco Research.

[B40-behavsci-15-00980] Kotz D., Brown J., West R. (2013). Predictive validity of the Motivation To Stop Scale (MTSS): A single-item measure of motivation to stop smoking. Drug and Alcohol Dependence.

[B41-behavsci-15-00980] Lerman C., Gold K., Audrain J., Lin T. H., Boyd N. R., Orleans C. T., Wilfond B., Louben G., Caporaso N. (1997). Incorporating biomarkers of exposure and genetic susceptibility into smoking cessation treatment: Effects on smoking-related cognitions, emotions, and behavior change. Health Psychology.

[B42-behavsci-15-00980] Lerman C., Kash K., Stefanek M. (1994). Younger women at increased risk for breast cancer: Perceived risk, psychological well-being, and surveillance behavior. Journal of the National Cancer Institute. Monographs.

[B43-behavsci-15-00980] Li W., Song L. Q., Tan J. (2014). Combined effects of CYP1A1 MspI and GSTM1 genetic polymorphisms on risk of lung cancer: An updated meta-analysis. Tumor Biology.

[B44-behavsci-15-00980] Lipkus I. M., Eissenberg T., Schwartz-Bloom R. D., Prokhorov A. V., Levy J. (2014). Relationships among factual and perceived knowledge of harms of waterpipe tobacco, perceived risk, and desire to quit among college users. Journal of Health Psychology.

[B45-behavsci-15-00980] Lipkus I. M., McBride C. M., Pollak K. I., Lyna P., Bepler G. (2004). Interpretation of genetic risk feedback among African American smokers with low socioeconomic status. Health Psychology.

[B46-behavsci-15-00980] Magnan R. E., Koblitz A. R., Zielke D. J., McCaul K. D. (2009). The effects of warning smokers on perceived risk, worry, and motivation to quit. Annals of Behavioral Medicine.

[B47-behavsci-15-00980] Marteau T. M., French D. P., Griffin S. J., Prevost A. T., Sutton S., Watkinson C., Attwood S., Hollands G. J. (2010). Effects of communicating DNA-based disease risk estimates on risk-reducing behaviours. Cochrane Database of Systematic Reviews.

[B48-behavsci-15-00980] McBride C. M., Koehly L. M., Sanderson S. C., Kaphingst K. A. (2010). The behavioral response to personalized genetic information: Will genetic risk profiles motivate individuals and families to choose more healthful behaviors?. Annual Review of Public Health.

[B49-behavsci-15-00980] Nair S. S., Chakravarty D., Dovey Z. S., Zhang X., Tewari A. K. (2022). Why do African-American men face higher risks for lethal prostate cancer?. Current Opinion in Urology.

[B50-behavsci-15-00980] Pereira L., Mutesa L., Tindana P., Ramsay M. (2021). African genetic diversity and adaptation inform a precision medicine agenda. Nature Reviews Genetics.

[B51-behavsci-15-00980] Prochaska J. O., Johnson S., Lee P., Shumaker S. A., Ockene J., Riekert K. A. (2009). The transtheoretical model of behavior change. The handbook of health behavior change.

[B52-behavsci-15-00980] Ramsey A. T., Bourdon J. L., Bray M., Dorsey A., Zalik M., Pietka A., Salyer P., Chen L., Baker T. B., Munafò M. R., Bierut L. J. (2021). Proof of concept of a personalized genetic risk tool to promote smoking cessation: High acceptability and reduced cigarette smoking. Cancer Prevention Research.

[B53-behavsci-15-00980] Reyna V. F., Nelson W. L., Han P. K., Dieckmann N. F. (2009). How numeracy influences risk comprehension and medical decision making. Psychological Bulletin.

[B54-behavsci-15-00980] Roberts M. E., Susswein L. R., Janice Cheng W., Carter N. J., Carter A. C., Klein R. T., Hruska K. S., Marshall M. L. (2020). Ancestry-specific hereditary cancer panel yields: Moving toward more personalized risk assessment. Journal of Genetic Counseling.

[B55-behavsci-15-00980] Rosenstock I. M. (1966). Why people use health services. The Milbank Memorial Fund Quarterly.

[B56-behavsci-15-00980] Schwartz A. G., Cote M. L., Wenzlaff A. S., Land S., Amos C. I. (2009). Racial differences in the association between SNPs on 15q25.1, smoking behavior, and risk of non-small cell lung cancer. Journal of Thoracic Oncology.

[B57-behavsci-15-00980] Sheeran P., Harris P. R., Epton T. (2014). Does heightening risk appraisals change people’s intentions and behavior? A meta-analysis of experimental studies. Psychological Bulletin.

[B58-behavsci-15-00980] Siegel R. L., Miller K. D., Jemal A. (2017). Cancer statistics, 2017. CA: A Cancer Journal for Clinicians.

[B59-behavsci-15-00980] Sloan M. E., Gowin J. L., Yan J., Schwandt M. L., Spagnolo P. A., Sun H., Hodgkinson C. A., Goldman D., Ramchandani V. A. (2018). Severity of alcohol dependence is associated with the fatty acid amide hydrolase Pro129Thr missense variant. Addiction Biology.

[B60-behavsci-15-00980] Stepler K. E., Gillyard T. R., Reed C. B., Avery T. M., Davis J. S., Robinson R. A. S. (2022). ABCA7, a genetic risk factor associated with Alzheimer’s disease risk in African Americans. Journal of Alzheimer’s Disease.

[B61-behavsci-15-00980] Sung Y. J., Winkler T. W., de Las Fuentes L., Bentley A. R., Brown M. R., Kraja A. T., Schwander K., Ntalla I., Guo X., Franceschini N., Lu Y., Cheng C., Sim X., Vojinovic D., Marten J., Musani S. K., Li C., Feitosa M. F., Kilpeläinen T. O., Chasman D. I. (2018). A large-scale multi-ancestry genome-wide study accounting for smoking behavior identifies multiple significant loci for blood pressure. American Journal of Human Genetics.

[B62-behavsci-15-00980] Tallis F., Eysenck H. J. (1994). Worry: Mechanisms and modulating influences. Behavioural and Cognitive Psychotherapy.

[B63-behavsci-15-00980] Thomas N. S., Salvatore J. E., Gillespie N. A., Aliev F., Ksinan A. J., Dick D. M., Spit for Science Working Group (2021). Cannabis use in college: Genetic predispositions, peers, and activity participation. Drug and Alcohol Dependence.

[B64-behavsci-15-00980] Wassenaar C. A., Conti D. V., Das S., Chen P., Cook E. H., Ratain M. J., Benowitz N. L., Tyndale R. F. (2015). UGT1A and UGT2B genetic variation alters nicotine and nitrosamine glucuronidation in european and african american smokers. Cancer Epidemiology, Biomarkers & Prevention.

